# Development of interventions for an intelligent and individualized mobile health care system to promote healthy diet and physical activity: using an intervention mapping framework

**DOI:** 10.1186/s12889-019-7639-7

**Published:** 2019-10-17

**Authors:** Yuling Chen, Fangqin Wu, Ying Wu, Jia Li, Peng Yue, Ying Deng, Karen V. Lamb, Simon Fong, Yisi Liu, Yan Zhang

**Affiliations:** 1 School of Nursing, Capital Medical University, 10 You-an-men Wai Xi-tou-tiao, Feng-tai District, Beijing, 100069 China; 2 Department of Adult Health Gerontological Nursing Rush University IL, Chicago, CA 60613 USA; 3 Department of Computer and Information Science, University of Macau, Macau, China

**Keywords:** Lifestyle, Behavior, Diet, Physical activity, Mobile health, Intervention mapping, Coronary heart disease, Behavior change

## Abstract

**Background:**

The mortality of coronary heart disease can be largely reduced by modifying unhealthy lifestyles. However, the long-term effectiveness of interventions for modifying unhealthy diet and physical inactivity of patients with coronary heart disease remain unsatisfactory worldwide. This study aims to systematically design a set of theory-based and evidence-based, individualized, and intelligent interventions for promoting the adoption and maintenance of a healthy diet and physical activity level in patients with coronary heart disease.

**Methods:**

The interventions will be delivered by a mobile health care system called **I**ndividualized, **I**ntelligent and **I**ntegrated **C**ardiovascular **A**pplication for **R**isk **E**limination. Three steps of the intervention mapping framework were used to systematically develop the interventions. Step 1: needs assessment, which was carried out by a literature review, in-depth interviews and focus group discussions. Step 2: development of objective matrix for diet and physical activity changes, based on the intersection of objectives and determinants from the Contemplation-Action-Maintenance behavior change model. Step 3: formulation of evidence-based methods and strategies, and practical applications, through a systematic review of existing literature, research team discussions, and consultation with multidisciplinary expert panels.

**Results:**

Three needs relevant to content of the intervention, one need relevant to presentation modes of the intervention, and four needs relevant to functional features of the application were identified. The objective matrix includes three performance objectives, and 24 proximal performance objectives. The evidence-based and theory-based interventions include 31 strategies, 61 evidence-based methods, and 393 practical applications.

**Conclusions:**

This article describes the development of theory-based and evidence-based interventions of the mobile health care system for promoting the adoption and maintenance of a healthy diet and physical activity level in a structured format. The results will provide a theoretical and methodological basis to explore the application of intervention mapping in developing effective behavioral mobile health interventions for patients with coronary heart disease.

**Trial registration:**

Chinese Clinical Trial Registry: ChiCTR-INR-16010242. Registered 24 December 2016. http://www.chictr.org.cn/index.aspx

## Background

The mortality of coronary heart disease (CHD) and the prevalence of cardiovascular major risk factors, such as hypertension, dyslipidemia, and diabetes [1, 2], can be largely reduced by modifying unhealthy lifestyles [3–6]. It has been suggested that lifestyle modification can reduce at least 64% of CHD-related mortality [7] and control 70% of cardiovascular major risk factors [4]. However, the prevalence of unhealthy lifestyles, such as unhealthy diet and physical inactivity, is still high both in China [8, 9] and around the world [4, 10, 11]. Recent large, nation-wide surveys found that more than 82% of Chinese adults have unhealthy diets [9], and 77% have low physical activity levels [8]. Furthermore, large international studies (PURE [12] and EUROASPIRE IV [10]) show that the prevalence rates of unhealthy diet and physical inactivity in CHD patients (including patients with acute coronary syndrome and those who had percutaneous coronary intervention [PCI]) remain more than 61 and 60%, respectively.

Previous studies for healthy diet and physical activity changes were carried out through group sessions [13], one-on-one face-to-face interviews [14, 15], or telephone counseling [16]. Despite the fact that these interventions have been successful in short-term and medium-term follow-up, a large proportion of CHD patients still had unhealthy diets and low physical activity in long-term follow-up [13, 14]. A recent large international multicenter study (EUROACTION [14]) found that 45 and 46% of CHD patients who had one-year face-to-face intervention still had unhealthy diet and low physical activity. Similarly, a large Community Interventions for Health (CIH) study found that in Chinese adults who had a two-year community-based multilevel intervention, beneficial changes were only noticed in physical activity but not diet [17].

Several barriers prevent the traditional interventions from achieving successful results. Firstly, many interventions that are targeted at changing unhealthy diet and physical inactivity are developed without following a systematic framework [18–20]. Secondly, the evidence underlying these interventions is not well grounded [20]. Without robust supporting evidence from previous studies, the quality of those interventions is doubtful [21–23]. Thirdly, evidence from a meta-analysis [24] shows that traditional interventions have generally been guided by continuum behavioral change theories, such as Social Cognitive Theory [25] and Theory of Planned Behavior [26]. However, those theories [25, 26] may be inappropriate in guiding the development of interventions which are targeted at the maintenance of healthy diet and physical activity for CHD patients, because those theories typically do not account for the post-intentional phase [20, 27, 28]. Finally, it has been argued that most traditional interventions do not provide patients with individualized interventions and real-time feedback [15]. When real-time feedback is provided, it requires intense use of healthcare resources [29]. Consequently, real-time feedback is criticized as unrealistic in clinical practice and therefore unsustainable [29].

To overcome those barriers, several interventions have been designed based on the Intervention Mapping (IM) framework [21, 30], which facilitates a stepwise process to develop, implement, and evaluate interventions in a systematic way [31]. Application of this framework has increased the likelihood of effectiveness of interventions regarding diet and physical activity [21, 32, 33]. A major strength of using the IM framework includes the possibility of selecting the most likely effective evidence-based methods and behavior change theories [34]. The Contemplation-Action-Maintenance Model (CAM), an integrated behavioral change model, which was developed based on the Health Action Process Approach (HAPA) [35] specifically targeting CHD patients, may serve as an appropriate behavior change theory for the development of interventions regarding the adoption and the maintenance of healthy diet and regular physical activity in CHD patients (Yue P, et al: Contemplation-action-maintenance model: Exploring the action and maintenance of health behavior among patients with coronary heart disease in the real world, Submitted). The advantage of the CAM model is that it clearly describes the roles of moderators and mediators during all stages of behavior change (including the motivational and volitional processes). This advantage implies that some supervision is provided during the motivational and volitional processes for effective interventions.

In addition to the evidence-based and theory-based approach, individualized and practical interventions that promote a healthy diet and physical activity with real-time feedback are required [36]. Mobile health (mHealth) technology is expected to fulfill those requirements [37–41]. However, just-in-time adaptive interventions have rarely been applied in existing mHealth interventions [36, 42, 43]. Many current mHealth interventions for diet and physical activity changes were developed without following a systematic approach [44, 45], or only deliver predefined interventions, which do not adaptively meet the individual’s needs and characteristics well [44, 46]. Consequently, although mHealth delivered interventions that target diet and physical activity have shown positive results, effect sizes of those interventions are often small [47–49].

We therefore aimed to develop an **I**ndividualized, **I**ntelligent and **I**ntegrated **C**ardiovascular **A**pplication for **R**isk **E**limination (**iCARE**) management system to increase adherence to a beneficial lifestyle (including healthy diet and physical activity). This article aims to describe how the IM framework and CAM behavior change model were used to develop a set of theory-based and evidence-based iCARE interventions to promote the adoption and maintenance of healthy diet and regular physical activity in patients with CHD.

## Methods

### Framework for intervention development

The first three steps of the IM framework [31] were used to develop the iCARE interventions in this study, which include: (1) needs assessment; (2) development of a matrix of objectives for diet and physical activity changes; and (3) formulation of evidence-based methods and strategies, and practical applications (According to Bartholomew et al. [31], practical application is defined as the specific translation of an evidence-based method for use in a way that fits the intervention population and the context in which the intervention will be conducted). We did not include steps 4–6 of the IM framework because they are related to the implementation, evaluation and sustainability of the intervention. Steps 4–6 will be reported separately. The iCARE interventions reported here are in accordance with the *Template for Intervention Description and Replication* (TIDieR) checklist [50].

### Step 1: needs assessment

According to IM framework, the aim of the needs assessment is to identify the factors that cause or influence the health problem that will be the focus of the intervention [31]. In China, it is estimated that 11 million patients suffer from CHD and the prevalence and mortality of CHD is continuously rising [51]. As diet and physical activity are key modifiable risk factors for CHD, the *2016 European Guidelines on Cardiovascular Disease Prevention in Clinical Practice* [1] and *AHA/ACCF Secondary Prevention and Risk Reduction Therapy Guidelines for Patients with Cardiovascular Disease* [52] recommend that all CHD patients adopting a healthy diet and physical activity level. However, there are several barriers such as emotions, psychological and spiritual beliefs, knowledge, social support, and cost that make it difficult to change diet and physical activity levels [53–56]. Consequently, even for patients who undertake PCI or have acute myocardial infarction, the prevalence of unhealthy diet and physical inactivity is still high [4, 10, 11]. Therefore, diet and physical activity were selected as target behaviors in this study.

Since the interventions will be delivered by an mHealth iCARE system (includes a specific application for CHD patients), we focus on assessing patients’ needs that are relevant to the content of the mHealth intervention (i.e. knowledge about healthy lifestyles and methods to maintain healthy lifestyles), presentation modes of the mHealth intervention (i.e. voice, video), and functional features (i.e. providing reminders) of the application (App). The needs assessment was carried out by means of a literature review, in-depth interviews and focus group discussions of CHD patients.

#### Literature review

A literature review was conducted to identify patients’ opinions and preferences about mHealth behavioral interventions. Both quantitative studies and qualitative studies were included in the literature review. The inclusion criteria for literature review were studies which: (1) were based on mHealth interventions; (2) targeted at health behaviors (including physical activity or diet); and (3) included patients with CHD. Studies which did not describe patients’ needs, opinions, or preferences about the mHealth intervention were excluded.

#### In-depth interview and focus group discussion

After approval by the institutional review committees of the Capital Medical University (No.2015SY45), semi-structured in-depth interviews and focus group discussions were conducted during August 2017, by a trained qualitative researcher (PY, female, PhD, associate professor) who is interested in health behavior change. Purposive sampling was used to select hospitalized CHD patients from two hospitals (Xuanwu Hospital and Beijing Chaoyang Hospital) affiliated with Capital Medical University in Beijing, China. The inclusion criteria for the in-depth interviews and focus group discussions were patients who: (a) had a documented diagnosis of CHD including acute myocardial infarction, unstable angina, or had primary or elective PCI; (b) used a smartphone daily; (c) agreed to participate; and (d) were 18 years or older. The exclusion criteria were patients who: (1) were unable to speak Mandarin; (2) had impaired hearing bilaterally; and (3) suffered from severe medical conditions (such as New York Heart Association classification IV). For the interviews, we used an interview guide (Additional file 1), which contained open-ended questions targeting patients’ needs that were initially drafted by a trained qualitative researcher (PY) and revised by the research team according to the study aim. After the aim and process of the interviews were introduced to patients by staff nurses, interviews were conducted in a quiet room by face-to-face at the hospitals until theoretical saturation was observed. Saturation was identified when the coding team (PY, YLC, YW) agreed that no new relevant knowledge was being obtained from new participants. In total, six CHD patients were interviewed individually, and eight CHD patients were involved in two focus group discussions (four for each focus group). Two patients (one for each focus group) dropped out during the focus group discussion because of the treatment in the hospital. With consent from participants, all interviews were audio recorded and field notes were made during the individual interview and focus group discussion. The average duration of individual interviews and focus group discussions were 49.8 min (39–60) and 135 min (120–150), respectively. All interviews were analyzed subsequently using inductive content analysis [57]. NVivo 10.0 was used to manage the data. All themes were derived from the data. The interviews were in accordance with the consolidated criteria for reporting qualitative research (COREQ) checklist [58].

#### Reasons of choosing CAM model to guide the design of the intervention

According to the TIDieR checklist [50], the description of the theory that underpins an intervention is needed to adequately explain its essential elements. The task of the first step of IM framework also includes the identification of an appropriate theory to guide the development of the intervention [31]. It was reported that for patients who have been diagnosed with CHD, the main challenge is building on volition to maintain a healthy diet and physical activity, with less effort on developing intention and building motivation for an action (taking action to change unhealthy behavior). However, the previously-mentioned behavioral change theories focused more on the process of promoting changes from intention to action taking and less on building volition to maintain healthy behaviors.

**Fig. 1:**
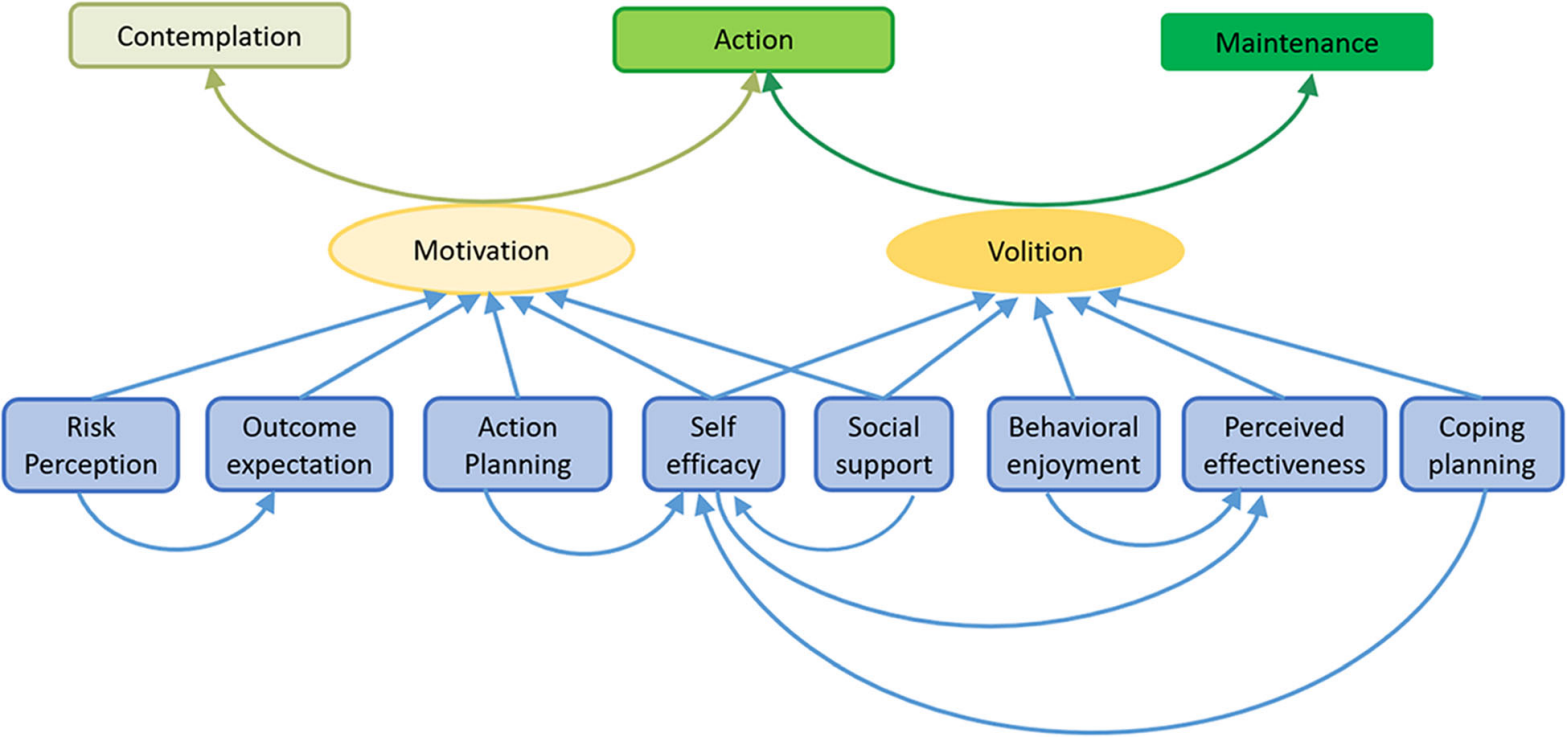
Contemplation-Action-Maintenance (CAM) model

The CAM model, a model that focuses on the motivation building and volition building processes in accordance with the specific behavior change stages of individuals, is a good fit to guide the development of interventions targeting healthy diet and physical activity in patients with CHD (Fig. 1). Briefly, the CAM model, is a behavioral change theory that uses the HAPA model [35] as the basic structure, and focuses on the characteristics of different stages of behavior change. According to the CAM model, higher levels of motivation help individuals move from building intention to taking action. After the action has been taken, sustaining the action depends on the strength of the volition. When the volition increases to a certain level, changed behavior can be maintained. In other words, motivation mediates the change from contemplation to action, while volition mediates the change from action to maintenance. The CAM model clearly outlines the following relationship: (1) risk perception, outcome expectation, action planning, social support, and action self-efficacy moderate motivation changing or building; and (2) behavioral enjoyment, effectiveness perception, coping planning, maintenance self-efficacy, and social support moderate volition changing or building. Therefore, the CAM model was chosen to guide the design of the theory-based interventions that enhance the achievement of long-term maintenance of healthy lifestyle.

### Step 2: development of a matrix of objectives for diet and physical activity change

The objective matrix was created by the intersection of objectives and determinants. The objectives include research objectives, performance objectives, and proximal performance objectives. Research objectives, which were formulated based on the needs assessment, are the overall objectives of the intervention and are used to guide the performance objectives and proximal performance objectives. Performance objectives, which are created based on the CAM model, are the behavioral outcomes to be achieved across the stages of change. Proximal performance objectives, which are generated by considering of the determinants of each performance objective, are the specific actions to be taken to facilitate the achievement of performance objectives. The determinants are factors that influence behavior change. In our work, both mediators and moderators in the CAM model serve as the determinants.

As mentioned earlier, the overall research objectives of the iCARE intervention are to reduce CHD mortality by promoting change and maintenance of healthy diet and physical activity. To keep the intervention simple and minimize burden on participants for dietary modification, we focused only on the important contributing factors to CHD [1] such as intake of salt and saturated fat instead of all recommendations for a healthy diet. The recommended goals for a healthy diet and physical activity level in this study followed the *Chinese Guidelines on Rehabilitation and Secondary Prevention of Coronary Heart Disease* [59] and the *2016 European Guidelines on Cardiovascular Disease Prevention in Clinical Practice* [1]. These recommendations include, a daily intake of no more than 5 g of salt [1, 59] and 25 g of cooking oil [59]; the saturated fat should be reduced to a maximum of 10% of total calories [1]; the accumulated physical activities should be at least 30 min per day, five days per week at moderate intensity (i.e. 150 min/week) or 15 min per day, 5 days per week at vigorous intensity (75 min/week), or a combination of both [1].

### Step 3: formulation of evidence-based methods and strategies, and practical applications

#### Defining criteria for the selection of evidence-based methods

In order to meet the study objectives and to design potentially effective interventions, a set of criteria for choosing an evidence-based method is required [33]. In our case, two types of criteria for the selection of evidence-based methods were developed: (1) general criteria, to identify existing effective and practical evidence-based methods potentially appropriate for patients with CHD; and (2) desirable criteria, to enable us to meet patients’ needs. Initial selection criteria were developed based on the results of the needs assessment and discussions with the research team. The criteria were then finalized by consensus after consultation with a multidisciplinary expert panel (MEP).

#### Reviewing existing evidence-based methods and strategies

Effective evidence-based methods and strategies of diet and physical activity for CHD patients were identified by systematically searching the PubMed database for relevant literature published between 1990 and 2016. The search strategy included the following medical subject heading terms in various combinations: chronic disease management, coronary heart disease, life style, health behaviors, diet, physical activity, sedentary lifestyle, intervention, clinical trial, and randomized controlled trial. The details of the search strategy are summarized in Additional file 2. Searches were not limited by publication type or language. The quality of the study and the effect size of the interventions were assessed during the literature review. Quality of the studies was evaluated using the Jadad scale [60] by two independent researchers (YLC and JL). If disagreement regarding a study arose, a third researcher (YD) additionally conducted an assessment. Only studies with a high level of evidence (above level V) and quality (Jadad scores above 3) were considered as potentially effective interventions. In addition, literature on the IM Taxonomy of Behavior Change Methods [20] and the Taxonomy of Behavior Change Techniques (BCT) [61] were reviewed to identify effective evidence-based methods from these taxonomies. Barriers related to the maintenance of healthy diet and regular physical activity and corresponding solutions were also identified in the literature review.

#### Selecting effective evidence-based methods and strategies

Several research team discussions and a round of consultation with the MEP were held to select the most likely effective evidence-based methods and strategies derived from the literature review based on the aforementioned criteria.

Firstly, the identified methods and strategies were initially screened independently against the general criteria by two researchers (YLC and JL) whose research area is the secondary prevention of cardiovascular disease and excluded if they did not meet the general criteria. The desirable criteria were then used as the reference to rank those selected methods and strategies in the initial screening. Individual ranking outcomes were then shared and discussed with the research team until a consensus was reached. After obtaining approval of the research team, the selected methods and strategies were matched with the matrix of change objectives to formulate a draft repository of evidence-based methods and strategies.

Secondly, a round of consultation with the MEP was held to evaluate the draft repository of evidence-based methods and strategies using the above general and desirable criteria. The MEP included a cardiovascular nurse, a community nurse, two cardiovascular physicians, and a lifestyle behavior psychologist. A consensus was required during the consultation with the MEP for methods or strategies to be included as potentially effective. Other possible effective methods and strategies or suggestions for changing unhealthy diet and physical inactivity were also summarized from this consultation with the MEP. The relevant barriers for maintaining healthy diet and regular physical activity as well as tailored solutions for the barriers identified in the literature review were also discussed during the consultation with the MEP.

#### Translating evidence-based methods into practical applications

Effective evidence-based methods and strategies which were selected in the above consultation with the MEP were then translated into practical applications by two researchers (YLC and FQW). To reach the goal of individualization of the interventions and aid the translation of the evidence-based methods into practical applications with technological features, we used the computer tailoring method [62] which is increasingly applied to develop effective interventions targeted at changing lifestyles [63, 64]. This method requires: (1) an intervention library; (2) a set of trigger rules based on IF-THEN algorithm; (3) media, for example, a video or cartoon, that delivers the individualized interventions to the specific patient; and (4) a data source [62].

Based on the computer tailoring method, a set of complex IF-THEN rules was designed to trigger the practical applications. These IF-THEN rules were composed of demographic characteristics, medical history, baseline assessment of lifestyle behaviors, dynamic assessment of lifestyle and cardiovascular major risk factors (i.e. salt intake, number of steps walked, blood pressure, blood glucose), stages of behavior change, and the levels of moderators and mediators from the CAM model. To effectively translate evidence-based methods into practical applications, we clearly described *what* (content), *when* (time to send), *how many* (dose), *how often* (frequency), *to who* (such as patient, nurse), and *by which media* (i.e. text, video, or cartoon) for each practical application.

#### Formulating of iCARE interventions

The practical applications developed were discussed by the research team until a consensus was reached. A second round of consultation with the MEP was then held to assess the feasibility of each practical application. During the second round of consultation with the MEP, we included 2 additional experts, a nutritionist and an athletic trainer. Suggestions for the implementation of the practical applications were also generated from this consultation with the MEP. After the assessment by the consultation with the MEP, a set of iCARE interventions to support healthy diet and physical activity changes were formulated.

## Results

### Needs assessment

Table 1 lists the patients’ needs derived from the literature review. Five needs relevant to intervention content, two needs relevant to intervention presentation modes, and eight needs relevant to functional features of the App were identified by the literature review. For the interviews, six CHD patients (mean age 47 years) were interviewed individually, and another six CHD patients (mean age 64 years) were involved in two focus group discussions (3 for each focus group). Patient demographics are shown in Additional file 3. The most frequent and common needs derived both from the literature review and interviews are described below.

**Table 1:** Needs assessment based on literature review

Topic	Summary of findings
Contents of the intervention	1. Providing food and exercise suggestions that are relevant, personalized, and actionable 2. Providing knowledge that healthy lifestyle and behavior change is clear, accurate, valid, and reliable 3. Specific physical activity plans 4. Follow-up tailored messages 5. Feedback about progress and individual barriers
Presentation modes of the intervention	1. Text message is presented in a variety font 2. Attention getting pictures and/or videos
Functional features of the app	1. Communication with doctors 2. Self-risk assessment 3. Tailored education 4. Blood pressure management 5. Health status recording/monitoring activity without user’s interaction 6. Reminders 7. Being active with friends or families 8. Data sharing ability

#### Patients’ needs of the content of mHealth intervention

##### Increasing knowledge of health

Knowledge of health refers to information that is relevant to lifestyle, symptoms and coronary heart disease. Three patients stated their opinion about knowledge of health:

*I had no idea of how to keep healthy after I had a PCI. Although I know that I should eat healthier, but I don’t know the specific knowledge about eating healthily after having PCI.* [P4, Male, 41 years old]

##### Making action plans

Action plans refer to the details of the context, frequency, duration and intensity of starting a behavior. Patients preferred simple, practical, individualized, and optional action plans. Patient 1 shared his perceptions about action plans:

*What I want from the App exactly is a simple and practical action plan based on my own health problem related data. I don’t like complicated information, I love simplicity. I prefer multiple action plans so that I can choose the most appropriate one. For instance, if my blood sugar keeps increasing for several days, then I want to receive a message like: ‘Your blood sugar has increased by 2 mmol/L recently. There are several options for you, such as eating less or walking 5 kilometers.’* [P1, Male, 34 years old]

#### Patients’ needs of the presentation modes of mHealth intervention

##### Presenting information in an attractive way

Information presented in attractive formats means that the information is interesting, customized, or eye-catching. Information presented in voice, videos, animations, with avatars, large font, humous tones, and muted color can effectively attract patients’ attention.

*I prefer information presented in large font, a humous tone, and muted color. Voice and video are more suitable for me.* [P2, Female, 52 years old]

#### Patients’ needs of functional features of the app for CHD

##### Continually monitoring of health

Monitoring of health refers to recording of lifestyle or cardiovascular major risk factors by patients or healthcare providers. Monitoring lifestyle and major cardiovascular risk factors via an easy way (such as a device) that does not take much effort is a common need.

*I need to monitor my blood pressure and blood glucose every day. I think a healthcare App should be linked to some other monitoring devices. It will be much more convenient if the device is directly connected to the App and the data is transferred to the App directly, so that I don’t need to input the blood glucose by myself.* [P1, Male, 34 years old]

##### Providing healthy reminders

Healthy reminders refer to messages that remind someone to eat healthy, exercise, and measure blood pressure, or blood glucose. Patients stated that providing multiple choices regarding the contents of the reminders, and the time and frequency for receiving the reminders are necessary.

*I think a reminder is particularly important for both young patients and old patients. Young patients like me are busy working, older people may not have a good memory. For me, reminding me to measure blood pressure on time is necessary.* [P1, Male, 34 years old]

##### Providing consulting services

Consulting services refer to access for patients to consult with doctors or nurses about their symptoms and concerns. Patients 2 and patient 5 revealed their experience as follow:

*One day, when I rested in bed after mountain climbing with my friends, my heart felt uncomfortable. I was afraid that I had a heart attack. I felt helpless at that time. If there was a doctor who could tell me that it was not a heart attack, I would feel at ease. I really hope the App has a service that I can use to consult with the doctors when I feel uncomfortable*. [P5, Male, 55 years old]

##### Communicating between patients

Communicating between patients means that patients share their similar experiences, symptoms, and feelings with each other. Patients stated that the communication between each other is a good way to understand the symptoms of the disease and to learn about healthy lifestyles.

*I think it is important that we (patients) can communicate with each other. For example, I do not know whether the slight pain in my heart is normal after the operation (PCI). When I talked to my wardmate, he said he also had the same feeling. Then I realized this is normal.* [P4, Male, 41 years old]

### Matrix of objectives for diet and physical activity changes

The matrix of objectives for diet and physical activity changes is displayed in Table 2. Based on the CAM model, three performance objectives were formulated to promote a healthy diet and regular physical activity including: (1) building intention to change; (2) enhancing motivation of taking action; and (3) enhancing volition of maintaining action. As shown in Table 2, a total of 24 proximal performance objectives were created: three for risk perception; two for outcome expectations; three for action self-efficacy; two for action planning; five for maintenance of self-efficacy; one for coping planning; one for behavioral enjoyment; one for effectiveness perception; and three for social support. Of all the established proximal performance objectives, ten are aimed at promoting the maintenance of the healthy lifestyles, such as: (1) increasing patients’ perceptions about the improvements in physiological indexes from diet and physical activity changing (mediator: effectiveness perception); and (2) promoting patients coping with the barriers of maintaining healthy diet and regular physical activity (mediator: coping planning).

**Table 2:** Matrix of objectives for diet and physical activity changes

Performance Objectives (PO)	Mediators	Moderators	Proximal performance objectives (PPOs)
PO1: Building intention to change	Motivation	Risk perception	PPO1: Promoting patients to identify cardiovascular risk factors
			PPO2: Increasing patients’ awareness of the adverse consequences related to these risk factors
			PPO3: Increasing patients’ awareness of the severity of coronary heart disease
		Outcome expectations	PPO4: Increasing patients’ perception of the benefits for adoption of healthy diet and regular physical activity
			PPO5: Increasing patients’ decisiveness and supporting them in establishing an intention to adopt healthy diet and regular physical activity
PO2: Building and enhancing motivation of action taking	Motivation	Action Self-efficacy	PPO6: Helping patients to uncover barriers of diet and physical activity changes
			PPO7: Increasing patients’ confidence in diet and physical activity changes through role-modeling
			PPO8: Increasing knowledge and skills related to healthy diet and physical activity
			PPO9: Correcting patients’ misunderstanding about changing unhealthy diet and physical inactivity
			PPO10: Helping patients to recognize their ability to change
			PPO11: Decreasing patients’ fear of difficulty in diet and physical activity changes
			PPO12: Increasing social support
		Action planning	PPO13: Making a specific and individualized action plan for diet and physical activity changes with patients, and ensuring patients confirm and accept it.
			PPO14: Promoting patients to implement the action plan
PO3: Building and enhancing volition of action maintaining	Volition	Maintenance Self-efficacy	PPO15: Increasing patients’ confidence in maintaining healthy diet and regular physical activity
			PPO16: Increasing patients’ perception of physiological responses
			PPO17: Helping patients to uncover the barriers in maintaining healthy diet and regular physical activity
			PPO18: Increasing patients’ perception of positive experience from healthy diet and regular physical activity changing
			PPO19: Increasing patients’ awareness of progress in healthy diet and regular physical activity
		Coping planning	PPO20: Promoting patients coping with the barriers of maintaining healthy diet and regular physical activity
		Behavioral enjoyment	PPO18: Increasing patients’ perception of healthy diet and regular physical activity induced positive experience
		Effectiveness perception	PPO21: Increasing patients’ perceptions about the improvements in physiological indexes from diet and physical activity changes
		Social support	PPO22: Increasing family-support
			PPO23: Increasing peer-support
			PPO24: Increasing professional-support

### Evidence-based methods, strategies, and practical applications formulated

#### Criteria for the selection of evidence-based methods defined

Three general criteria and 20 desirable criteria for selection of effective methods for diet and physical activity change were developed and are presented in Table 3. The three general criteria were: methods that are (1) related to diet and physical activity changes; (2) safe for the target patients; and (3) feasible to implement. The desirable criteria included simple, interactive, and enjoyable.

**Table 3:** Criteria for selection of effective methods for diet and physical activity changes

Inclusion criteria	Source
General criteria	
1. Focus on diet change or physical activity change	Research team discussion
2. Safe for the target patients (1) The intensity, frequency, content of the intervention should be certified by experts. (2) The intervention will not increase the rate of acute cardiovascular events. (3) Strategies to protect patients’ privacy are covered in the study.	Literature review, research team discussion
3. Feasible to implement (1) Affordable to implement. (2) Practical with limited human resources	Literature review, focus group
Desirable criteria	
4. With high effectiveness in diet change or physical activity change (1) internal validity: •Odds ratio > 0.8 •Effect size (r > 0.3 or d > 0.2) (2) external validity: Intervention were found to be effectiveness in many literatures	Literature review/expert consultation
5. Consider the circumstances	In-depth interview, focus group
6. Met patients’ needs	In-depth interview
7. Culturally appropriate	In-depth interview, literature review
8. More attention to life	In-depth interview
9. Simple	In-depth interview
10. Clear	In-depth interview
11. Easy to understand	In-depth interview
12. Appropriate for patients with coronary heart disease	In-depth interview
13. Enjoyable	In-depth interview
14. Personalized	In-depth interview, focus group, literature review
15. Considerate	In-depth interview
16. Accept interruption	In-depth interview
17. With incentive	In-depth interview
18. Visible	In-depth interview
19. Appropriate frequency	In-depth interview, focus group
20. Continuous	In-depth interview, focus group
21. Self-monitoring functions available	Literature review
22. Alert functions available	Research team discussion, literature
23. Interactive function available	In-depth interview, focus group, Literature review

#### Evidence-based methods and strategies selected

A total of 10,731 articles were initially identified through the database search, of which 769 were duplicates, and 9962 articles that were screened for eligibility. A total of 9080 were found irrelevant after screening the titles and abstracts, and 882 full-text articles were carefully reviewed. After reading the full texts, 151 articles relating to healthy diet and 181 articles relating to physical activity were finally included. Of the articles included, strategies or evidence-based methods were identified in 187 articles of which 77 articles related to improving unhealthy diets and 110 articles regarding improving physical inactivity. By integrating the results, a total of 48 strategies and 158 evidence-based methods were derived from the systematic review.

The above strategies and evidence-based methods were reviewed by the study researchers and evaluated during several discussions with the research team based on the established criteria as displayed in Table 3, after which 32 strategies and 63 evidence-based methods that met the criteria were selected. After a round of consultation with the MEP, 31 strategies and 61 evidence-based methods were finally included in the study (suggestions from the consultation with the MEP are presented in Additional file 4). The 31 strategies are displayed in Table 4. For example, to improve social support, the following strategies were found: peer support, role modelling, and family support. Examples of evidence-based methods for diet and physical activity change are displayed in Tables 5 and 6, respectively. For example, guided by the strategy ‘dynamic monitoring and individualized, immediate feedback’, two theory-based methods were identified: (1) monitor and provide feedback on performance (e.g. salt intake) of the behavior; and (2) monitor and provide feedback on the outcomes (e.g. blood pressure) of the behavior.

**Table 4:** Strategies for modifying diet and physical activity

Strategies		Strategies derived from BCT or IM taxonomy		Precontemplation Stage		Contemplation Stage				Action stage and Maintenance stage				Number of evidence-based methods (*N* = 61)
		BCT	IM Taxonomy	Risk perception	Outcome expectation	Action planning	Action self-efficacy	Coping self-efficacy	Coping planning	Behavioral enjoyment	Effectiveness perception	Social support	Maintenance self-efficacy	
1	Psychological cues			√	√		√	√	√				√	12
2	Visualization			√	√		√				√			6
3	Peer impact			√	√							√		5
4	Fear arousal		√	√	√									5
5	Dynamic monitoring and individualized, immediate feedback	√	√	√				√					√	2
6	Increase knowledge interestingly	√			√		√							2
7	Re-attribution	√	√		√									1
8	Personalized action planning	√				√								1
9	Friendly reminders					√								1
10	Role model/modelling		√				√	√				√	√	5
11	Authority influence						√							1
12	Comparison of behavior						√							1
13	Decisional balance						√							1
14	Easy-to-do						√							1
15	Encourage						√							1
16	Increase self-control						√							1
17	Self-affirmation		√				√							1
18	Self-reevaluation		√				√							1
19	Social support	√					√							2
20	Increase sense of control						√							3
21	Public commitment		√				√							1
22	Step by step						√							1
23	Experience of success or enjoyment	√						√		√			√	4
24	Accept							√					√	1
25	Increase sense of experience							√					√	3
26	Increase sense of gain							√					√	1
27	Increase sense of success							√					√	1
28	Individualization								√					1
29	Considerate service											√		1
30	Family impact											√		1
31	Stimulate interest													1

*BCT* Behavior Change Techniques, *IM* Intervention Mapping

**Table 5:** Matrix of strategies, theory-based methods, practical applications of changing unhealthy diet: examples

Phases of change	POs	Mediators	Moderators	PPOs	Strategies	Theory-based Methods	IF	AND	THEN: Practical applications	Mode
Precontemplation	PO1	Motivation	Risk Perception	PPO1	Dynamic monitoring and individualized, immediate feedback	Monitor and provide feedback on performance (e.g. salt intake) of the behavior. Monitor and provide feedback on the outcome (e.g. blood pressure) of the behavior.	Salt intake >5 g/d	Cooked soil intake < 25 g/d	Mr. Wang, we found that you have very good control of your lipid and cholesterol levels, which is good for your health. Your health would improve if you decreased your sodium intake, because a salty diet may increase the risk of developing hypertension.	Text- messaging with trend graph
Contemplation	PO2	Motivation	Action Planning	PPO13	Personalized action plan	Provide a personalized action plan based on the patient’s health condition and preferences. Patient can modify the action plan if they disagree with the plan.	Salt intake >5 g/d and Cooked oil intake > 25 g/d	Have an intention to change	Mr. Wang, here is the action plan we recommend for you to maintain a healthy diet. What do you think of it? If you accept it, please click the accept button. If not, you can click the edit button to change the plan and then submit it.	Text-messaging with a link to review action plan
Action	PO3	Volition	Self-Efficacy	PPO17	Psychological cues	Psychometric tests: To uncover the obstacles in the maintenance of healthy behavior change and to provide tailored feedbacks and suggestions.	Salt intake >5 g/d and Soil intake > 25 g/d	Action plan has been made	If you have a dietary action plan, but you can’t stick to it, please complete this questionnaire to tell us your experience.	Text-messaging with a link to Psychological test
Maintenance	PO3	Volition	Social Support	PPO23	Role model	Patients who maintain healthy behavior for more than 6 months, will serve as role models. Regular group discussions will be facilitated by the role models for other patients who encounter difficulties in the process of behavior change to help them learn coping strategies	Salt intake >5 g/d and Soil intake > 25 g/d	Maintaining healthy diet for 6 months or above	The topics of the group discussions are: (1) experiences shared by the role model, and (2) the role model answering the questions from other patients.	Group discussion in the app

PO: Performance Objectives; PO1: Building intention to change; PO2: Building and enhancing motivation of action taking; PO3: Building and enhancing volition of action maintaining; PPO: Proximal performance objectives; PPO1: Promoting patients to identify CHD risk factors; PPO13: Making a specific and individualized action planning of diet and physical activity changes for patients, and make sure patients confirm and accept it; PPO17: Helping patients to uncover the barriers in maintaining healthy diet and regular physical activity; PPO23: Increasing peer-support

**Table 6:** Matrix of strategies, theory-based methods, practical applications of changing physical inactivity: examples

Phases of change	POs	Mediators	Moderators	PPOs	Strategies	Evidence-based Methods	IF	AND	THEN-Interventions	Mode
Precontemplation	PO1	Motivation	Risk Perception	PPO2	Dynamic monitoring and individualized, immediate feedback	Monitor and provide feedback on performance (e.g. salt intake) of the behavior. Monitor and provide feedback on the outcome (e.g. blood pressure) of the behavior.	PA frequency < 5 times a week or PA time < 15 min a day or patients’ steps < 6500 steps a day	Fasting blood glucose > 7.0 mmol/L or arbitrary blood glucose > 11.1 mmol/L	Mr. Wang, You have a bad control in blood glucose at present. Regular physical activity can effectively reduce blood glucose. Do you know the risk of Hyperglycemia for coronary heart disease? This video will tell you.	Video
Contemplation	PO2	Motivation	Self-Efficacy	PPO7	Role model	Use the story of a role model who has healthy behaviors	PA frequency < 5 times a week or PA time < 15 min a day or patients’ steps < 6500 steps a day	Making a specific and individualized PA action plans	Do you remember Mr. Zhang, he was in the hospital near your bed in the same ward, whose illness and age are similar to yours? Mr. Zhang has kept a regular PA. His blood pressure and blood glucose level are now normal. We believe you could do equally well. Keep it up!	Text with voice
Action	PO3	Volition	Coping Planning	PPO20	Individualization	Provide personalized coping strategies according to barriers patients meet during the process of behavior change	Regular PA for several days but patients’ steps < 6500 steps today	Making a specific and individualized PA action planning	Mr. Wang, your physical activity report shows that you have done well in recent days. But you did not make much progress today. I guess there were some causes for this. Please answer the questionnaire and so we can try to understand the reason that you did not keep up with your regular physical activity.	text-messaging and a link to a questionnaire
Maintenance	PO3	Volition	Behavioral enjoyment	PPO18	Experience of success or enjoyment	Material rewards will be given to the participants after each goal is completed, such as scores, small red packets (or coupons) which can be exchanged for gifts.	PA frequency > 5 times a week and PA time > 15 min a day or patients’ steps > 6500 steps a day	Maintaining regular PA for 6 months or above	Scores + 60, Rank + 3, monetary incentives sent at random	System setting

POs: Performance Objectives; PO1: Building intention to change; PO2: Building and enhancing motivation of action taking; PO3: Building and enhancing volition of action maintaining; PPOs: Proximal performance objectives; PPO2: Increasing patients’ awareness of the adverse consequences related to these risk factors; PPO7: Increasing patients’ confidence in diet and physical activity changes through role-models; PPO20: Promoting patients to coping with the barriers in maintaining healthy diet and regular physical activity; PPO18: Increasing patients’ perception of healthy diet and regular physical activity induced positive experience; PA: physical activity

In addition, 19 barriers related to the maintenance of healthy diet and 15 barriers for maintaining regular physical activity and related solutions identified in the literature were also formulated in this consultation with the MEP (Additional file 5).

#### Practical applications developed

Based on the identified evidence-based methods and strategies, a total of 253 and 140 practical applications regarding diet and physical activity respectively were finally formulated. Examples of practical applications for diet and physical activity changes are displayed in Tables 5 and 6, respectively. Each practical application is based on a proximal performance objective and is triggered by a set of IF-THEN algorithms. The intervention message starts with the patients’ name. Take patient “Mr. Wang” for example, IF “Mr. Wang’s physical activity was in the action stage, and the patient has walked < 6500 steps during the day”, THEN the text-message and a link to a questionnaire is sent: “Mr. Wang, your physical activity report shows that you have done well in recent days, but you did not make much progress today. I guess there were some causes for this. Please answer the questionnaire, so we can try to understand the reason that you did not keep up with your regular physical activity”. IF “the patient chooses the cause: I could not go out to do exercise because of the bad weather”, THEN a coping plan is sent: “Please, watch the video. You can find some indoor activities that may be suitable for you.”

To stimulate the interest of patients and help them meet their goals, we developed a set of videos, comics, and cartoons to educate them on the importance of fundamental health concepts. For example, IF “the patient was in contemplation stage for diet and she/he had a salt intake >5 g/d, or/and cooking oil intake > 25 g/d, and a low level of outcome expectation”, THEN a message is sent that reads “Mr. Wang, do you want to know the benefits of low salt and low-fat diets? Please go watch the comic.” (refer to Additional file 6 for example of the comic).

## Discussion

The detailed systematic process for the development of theory-based and evidence-based iCARE interventions that targeted the adoption and maintenance of healthy diet and physical activity among patients with CHD is reported in this article. Following the IM framework and based on the CAM model, interventions in this study integrated patients’ needs of mHealth interventions, existing effective evidence-based methods from high-quality studies, and perspectives from healthcare professionals. The process of designing such interventions may serve as an example of how theory-based and evidence-based behavioral mHealth interventions can be developed.

Formulating evidence-based and theory-based interventions to increase the likelihood of the effectiveness of interventions on changing diet and physical activity is one of the strengths of this study. Dietary patterns and physical activity are long-term established habits that are difficult to change, and interventions for changing them are always complex [65]. Although there are increasing numbers of interventions [66, 67] developed following the IM framework to potentially increase the intervention effectiveness, currently the underlying evidence base of those interventions is less grounded [20, 68]. Many of the previous attempts in choosing behavior change methods involved BCT technique [69], despite the fact that this technique is more suitable for intervention coding than intervention development [20]. Although useful, BCT technique has been criticized because this approach contains both ineffective and counter-effective methods [20]. According to IM taxonomy, an effective behavior change method should perform all the following actions: target behavioral changeable determinants that predict the relevant behavior; select evidence-based methods and carefully match them with behavior change determinants; and translate the evidence-based methods into a practical application in a way that fits with the target patients, culture, and context [20]. To increase the efficacy of the iCARE interventions, we carefully chose an integrated behavior change model, the CAM model, and used the mediators and moderators from this theory as behavioral determinants to guide the development of behavior change methods. More importantly, the selection of potential effective evidence-based methods was based on a systematic review of the existing literature and a rigorous evaluation of research quality. In addition, each evidence-based method was matched with one mediator to assure that they could in fact change behavior.

Utilizing mHealth technology to support the translation of evidence-based methods into practical applications is another strength of this study. Individualized interventions and real-time feedback for patients are key strategies for supporting diet and physical activity changes, and mHealth technology offers a platform for providing the strategies [41]. Existing mHealth interventions for diet and physical activity changes are not always specifically geared to the individual’s needs and characteristics [21]. To realize the potential of individualized intervention and to increase the likelihood of the effectiveness of the iCARE interventions, we developed evidence-based methods and strategies that took into account patients’ needs regarding mHealth interventions based on a literature review and patient interviews. In addition, we translated the evidence-based methods into practical applications using a computer tailoring method [62], which integrated multiple influencing factors (i.e. age, sex, health condition, behavior change stages, mediators and moderators) into a set of complex rules.

This mHealth-based intervention may have important implications for secondary prevention of CHD in China in particular. The incidence and mortality of CHD is increasing both in China [51] and around the world [3–6]. Reversal of this trend will require development of effective and feasible intervention designed to influence large numbers of CHD patients toward movement away from unhealthy lifestyles [7]. Using traditional face-to-face interventions does not reach large numbers of patients, and involves significant challenges such as time-consuming and high-costs [29]. The Stepathlon Cardiovascular Health Study showed that the implementation of mHealth-based lifestyle intervention was low-cost and was feasible on a large-scale in low- and middle-income countries [70]. Of specific concern is that without self-report of diet intake and physical activity levels, tailored intervention strategies cannot be used. To improve the feasibility of the intervention, devices such as a pedometer [70] and incentives to increase the adherence of self-report will be needed.

The study has several limitations. First, the efficacy of the intervention still has to be evaluated by a randomized controlled trial (the randomized control trial is ongoing). Second, only the PubMed database was searched in the systematic literature review. Although PubMed is one of the largest databases of biomedical literature [71], we acknowledge that some potentially useful interventions for diet and physical activity changes may have been overlooked. Third, the size of the focus groups for the needs assessment was limited to only three participants for each focus group, which is too small for focus group discussion based on the COREQ checklist [58]. However, by integrating the results of the literature review, individual interviews, and focus group discussions, we believe that these multifactorial needs assessments may be helpful in identifying the health problems and factors for the focus with the target population. Finally, we only focused on two important lifestyle factors, diet and physical activity, rather than including other factors such as smoking and alcohol intake. Further studies are needed to explore more comprehensive interventions for multiple lifestyles changes.

## Conclusion

This article describes the development of the iCARE interventions to promote the adoption and maintenance of a healthy diet and physical activity level in a structured format. The results of the research will provide a theoretical and methodological basis to explore the application of IM in developing effective behavioral mHealth interventions for patients with CHD.

## Supplementary information

**Additional file 1.** Topic guide for interviews.

**Additional file 2.** Searching strategies.

**Additional file 3.** Demographics of patients enrolled in the in-depth interviews and focus group discussions.

**Additional file 4.** Suggestions from the consultation with multidisciplinary expert panels.

**Additional file 5.** Barriers and solutions for the adoption and maintenance of healthy diet and physical activity among patients with coronary heart disease.

**Additional file 6.** Comic about the benefits of low salt and low fat.

## Data Availability

The datasets during and/or analyzed during the current study available from the corresponding author on reasonable request.

## Abbreviations

ACCF: American College of Cardiology Foundation
AHA: American Heart Association
App: Application
BCT: Behavior Change Techniques
CAM: Contemplation-Action-Maintenance
CHD: Coronary heart disease
COREQ: Consolidated criteria for reporting qualitative research
EUROASPIRE: European Action on Secondary and Primary prevention through Intervention to Reduce Events
HAPA: Health Action Process Approach
iCARE: Individualized, Intelligent and Integrated Cardiovascular Application for Risk Elimination
IM: Intervention mapping
MEP: Multidisciplinary expert panel
mHealth: Mobile health
PCI: Percutaneous coronary intervention
PURE: Prospective Urban Rural Epidemiologic
TIDieR: Template for Intervention Description and Replication
