# Supplementary material for: Development of interventions for an intelligent and individualized mobile health care system to promote healthy diet and physical activity: using an intervention mapping framework
Source: BMC Public Health. 2019 Oct 17;19:1311. doi: 10.1186/s12889-019-7639-7 (PMC6798431; doi:10.1186/s12889-019-7639-7)
Supplement: Supplementary file 1 — Additional file 1. Topic guide for interviews. [file 12889_2019_7639_MOESM1_ESM.docx]

**Additional file 1. Topic guide for interviews.**

| 1. **What kind of Apps do you download in your smartphone? Which one do you use frequently?** 2. **What are the reasons that you use these Apps frequently? What are the characteristics of these Apps that interest you?** 3. **Have you ever used health related Apps? What is your experience when you use them? Why?** 4. **Have you ever thought there is an App which may help you manage your heart disease?**  - What kind of characteristics that you find this APP useful so that you are willing to use it? - What kind of knowledge do you want to know about your heart disease? - What kind of presentation that you prefer for viewing your interested knowledge? Text messaging? Picture? Animation? Video? Voice? And Why? - (If video) Who do you like to read out the message for you? Why? - (If animation) What kind of image character would you want in the animation? Why? - What kind of reminder do you like? - During the day, when do you like to receive these messages? - What kind of language style do you like? Serious? Lively? Humorous? Warm and sweet?   **5. Do you have any other specific preference or suggestions for the App?** |
| --- |

App, Application.
