# Supplementary material for: Development of interventions for an intelligent and individualized mobile health care system to promote healthy diet and physical activity: using an intervention mapping framework
Source: BMC Public Health. 2019 Oct 17;19:1311. doi: 10.1186/s12889-019-7639-7 (PMC6798431; doi:10.1186/s12889-019-7639-7)
Supplement: Supplementary file 2 — Additional file 2. Searching strategies. [file 12889_2019_7639_MOESM2_ESM.docx]

**Additional file 2: Searching Strategies**

PubMed databases were searched using the following MeSH terms (chronic disease management or Diabetes or coronary heart disease or cardiac rehabilitation) AND (life style OR sedentary lifestyle OR health behaviors OR health behavior OR behavior, health OR patient compliance OR behavior modification OR behavior modifications OR modification, behavior OR modifications, behavior OR risk factor OR risk factors OR factor, risk OR factors, risk OR blood pressure OR hypertension OR diet OR diet records OR diet, vegetarian OR diet, sodium-restricted OR diet, reducing OR diet therapy OR nutrition assessment OR nutrition disorders OR cholesterol OR triglycerides OR hypercholesterolemia OR index, body mass OR body mass index OR obesity OR weight loss OR overweight OR body weight maintenance OR body weight OR walking OR physical activity OR physical activities OR activities, physical OR activity, physical OR exercise) AND (incentive OR incentives OR motivation OR volition OR will OR intention OR efficacy, self OR self-efficacy OR pleasure OR happiness OR enjoyment OR social support OR Support, Social or risk awareness or risk perception or outcome expectancies or outcome expectation or Action planning or Behavior experience or efficacy perception or effect perception or coping planning or Behavior change technology) AND (intervention OR controlled before after studies OR evidence based healthcare management OR Intervention Study or Clinical trial).
