# Supplementary material for: Development of interventions for an intelligent and individualized mobile health care system to promote healthy diet and physical activity: using an intervention mapping framework
Source: BMC Public Health. 2019 Oct 17;19:1311. doi: 10.1186/s12889-019-7639-7 (PMC6798431; doi:10.1186/s12889-019-7639-7)
Supplement: Supplementary file 3 — Additional file 3. Demographics of patients enrolled in the in-depth interviews and focus group discussions. [file 12889_2019_7639_MOESM3_ESM.docx]

**Additional file 3. Demographics of patients enrolled in the in-depth individual interviews and focus group discussions**

| Number | Type | Gender | Age (years) | Diagnosis | Duration for interview | Location |
| --- | --- | --- | --- | --- | --- | --- |
| P1 | ID1 | Male | 34 | MI | 1 hour | Hospital 1 |
| P2 | ID2 | Female | 52 | MI | 56 minutes | Hospital 1 |
| P3 | ID3 | Male | 41 | UA | 39 minutes | Hospital 1 |
| P4 | ID4 | Male | 41 | MI | 41 minutes | Hospital 1 |
| P5 | ID5 | Male | 55 | MI | 58 minutes | Hospital 1 |
| P6 | ID6 | Male | 55 | MI | 45 minutes | Hospital 2 |
| P7 | FG1 | Male | 44 | MI | 2.5 hours | Hospital 2 |
| P8 | FG1 | Male | 67 | MI | 2.5 hours | Hospital 2 |
| P9 | FG1 | Female | 75 | UA | 2.5 hours | Hospital 2 |
| P10 | FG2 | Female | 78 | MI | 2 hours | Hospital 2 |
| P11 | FG2 | Male | 55 | MI | 2 hours | Hospital 2 |
| P12 | FG2 | Male | 66 | MI | 2 hours | Hospital 2 |

MI, myocardial infarction; UA, unstable angina; P, patient; ID: in-depth interview; FG, focus group discussion.

**Needs assessment: main themes emerging from the in-depth interviews and focus group**

| **Themes** | **Characteristics** |
| --- | --- |
| 1. Reliability | Accurate-data |
|  | Privacy- protection |
|  | Respect |
|  | Directly-imported information |
| 1. Operability | To-do list |
|  | Simplified-Action Platform |
|  | Complete problem-solving |
| 1. Specialization | Professional content |
|  | Professional team |
|  | Professional health care |
| 1. Individualization | Personal regular pattern |
|  | Different design |
|  | Varies care provision |
|  | Explain personal issue |
| 1. Interactivity | Provide feedbacks by professional person |
|  | Patient-group discussion |
|  | Role model |
| 1. Flexibility | Target values should not be too stringent |
|  | Adopt possible change |
|  | Multi-choice |
|  | Objectives should be adjusted according to patients’ action |
| 1. Self-motivated | Less interruption |
|  | Do not control patients |
| 1. User-friendly design | Interesting |
|  | Rigorous &Understandable |
|  | Presentative design |
|  | Kind-remind |
|  | Remote diagnosis function |
|  | Focus on patients concerns |
|  | Convenient plus service |
| 1. Continuation | Recording |
|  | Consistent connecting |
|  | Regular updates |
| 1. Considerate | Consider patients’ health condition |
|  | Appreciate patients’ lived experience |
|  | Infrequent need to input data |
|  | Teach the users how to use the App |
| 1. Focused on patients’ experience | Respect patients’ feelings |
|  | Offer hope to patients  Relaxing |
|  | Respect patients’ starting point (lacking of non-professional knowledge) |
|  | Respect patients’ starting point (previous Unhealthy Behavior) |
|  | Close to patients’ lived experience |
|  | Respect patients' practical experience |
|  | Help patients to develop a habit to use the App |
|  | Meet multiple needs |
|  | Meet patients’ interest rather than compulsion |
|  | Increase patients’ risk awareness |
|  | Understand patients’ sense of-worth |
|  | Uncover the meaning of life of patients |
|  | Focus on the enjoyment for using the App |
|  | Focus on the psychological pressure of young and middle-aged patients |
|  | Language style is informal |
|  | Let patients feel to be concerned about |
|  | Language is warm and humorous |
|  | Be sincere in tone with the respect for the patients. |
|  | Unable to understand others’ difficulties |
|  | Don't use complex words. |
|  | Promote patients to cherish the opportunity of rebuilding healthy behavior |
|  | Provide information about death |
|  | Understanding the step-by-step changes in behaviors |
|  | Simpler is better |
|  | Multiple functions and increasing more function gradually |
|  | The intellectual solutions for problem |
|  | Offer basic service and enrich them gradually |
|  | Don't use frequent reminders |
| 1. Information with multiple presentation modes | Voice messages are more popular |
|  | Unlimited the form of messages |
|  | Unique ringtone |
|  | Different attitudes towards virtual image |
|  | Consideration about the relationship between universality and individualization |
|  | Time preference |
|  | Network flow |
|  | Animation format |
|  | Color preference |
