# Supplementary material for: Development of interventions for an intelligent and individualized mobile health care system to promote healthy diet and physical activity: using an intervention mapping framework
Source: BMC Public Health. 2019 Oct 17;19:1311. doi: 10.1186/s12889-019-7639-7 (PMC6798431; doi:10.1186/s12889-019-7639-7)
Supplement: Supplementary file 4 — Additional file 4. Suggestions from the consultation with multidisciplinary expert panels. [file 12889_2019_7639_MOESM4_ESM.docx]

**Additional file 4. Suggestions from the consultation with multidisciplinary expert panels**

| **Strategies or themes** | **Suggestions** |
| --- | --- |
| **Round One** | |
| Expert authority | 1. Increasing patients’ risk perception through authoritative cardiovascular experts |
| Peer influence | 2. Medical staff-led group discussion will not let patients relax. It is better to use peer-led group discussion. The key role of medical staff lies in evaluation.  3. Communication between peers is more important than communication with general people.  4. Ranking is very important. Comparing the ranking of peers by histogram or the gap between peer ranking and achievement of their own goals will motivate patients more. |
| Role model | 5. Behavior models are very suitable for middle-aged and elderly people. |
| Social support | 6. Social support or network support may be better than family support. |
| Reward | 7. It can be in the form of "stamping the red seal" or "exchanging integrals for trees". Praise can be replaced by integral system. |
| Emotional support | 8. Emotional support is more effective than physical encouragement. |
| Individualized demand | 9. Researchers ask patients whether they need reminders when they discharge from hospital. If patients choose "yes", then the App should provide reminders for them. In addition, special and tailor-made services are needed, such as booking an appointment for a doctor consultation.  10. It must be polarizable, so interventions should be patient-oriented. |
| Stage target | 11. External motivation disappears quickly, but internal motivation can be stimulated by a small goal. It must be a goal that patients can achieve. |
| Enjoyment | 12. For making the App more appealing, patients can choose a virtual character such as a cute animal as an avatar. The behavior or physical status of the animal reflect the patient's well-being state.  13. Design a game to increase physical activity (i.e. if you exercise, the kitten will look better; if you are angry, the kitten will be angry) |
| Update material | 14. It is necessary to update the material. |
| Appropriate frequency | 15. Don't overwhelm the patients with too much information each day. |
| User-friendly interface | 16. The menu of the App should be categorized. |
| Safety for physical activity | 17. Give priority to the patient's feelings and gradually increases the amount of exercise according to the guidelines.  18. For patients with post-AMI PCI (acute coronary syndrome), it must be the physician who develops the exercise prescription. Such patients (including those with heart failure) are generally at high risk.  19. PCI after non-AMI is generally a low-risk patient. It is necessary to contact the patients during patients’ hospitalization in order to gain a sense of trust and carry out risk assessment.  20. Monitoring oxygen consumption and oxygen saturation to ensure the safety of physical activity.  21. Sleep is also important for the rehabilitation of patients with CHD. Blood oxygen saturation can be measured by finger cuffs and apnea can be prevented by using dental pads and brackets.  22. Don't look at the patient's increment. Compare the patient's self-perception before and after, and take the patient's self-perception as the first place.  23. Be cautious about ACS patients, especially those after myocardial infarction. |
| **Round two** | |
| Diet assessment | 1. The dietary assessment scale needs to be quantified and correlated with coronary heart disease.  2. Food intake can be assessed by comparing food models.  3. The size of food intake can be assessed by measuring the distance by mobile phone.  4. The investigations of salt and oil intakes are difficult. It can be done by asking the patient how many grams of salt or oil are in a bag at home, and how long do they finish consumption of them?  5. Pay attention to the intakes of other high-salt condiments, or pickles. |
| Action Planning | 6. Accept that patients cannot change all unhealthy eating habits.  7. Express full respect for patients' eating habits.  8. Develop a simple and operational diet plan.  9. Give patients freedom of diet without restricting their dietary preferences. Therefore, detailed recipes are not recommended.  10. The dietary energy intervention plan should be formulated according to the patient's BMI. |
| Incentive method | 11. Using peer incentives to allow patients to manage themselves independently.  12. Let patients supervise each other and experts do not intervene them. |
| Method of attracting patients | 14. The patients in the group were given a green channel to enjoy preferential treatment.  15. Provide patients with practical and meaningful help that will truly benefit them.  16. Give medication guidance.  17. To give guidance on adverse reactions of exercise and taking medications.  18. Provide advisory channels.  19. Provide personalized services, such as nursing housekeeper. |
