# Supplementary material for: Development of interventions for an intelligent and individualized mobile health care system to promote healthy diet and physical activity: using an intervention mapping framework
Source: BMC Public Health. 2019 Oct 17;19:1311. doi: 10.1186/s12889-019-7639-7 (PMC6798431; doi:10.1186/s12889-019-7639-7)
Supplement: Supplementary file 5 — Additional file 5. Barriers and solutions for the adoption and maintenance of healthy diet and physical activity among patients with coronary heart disease. [file 12889_2019_7639_MOESM5_ESM.docx]

**Additional file 5. Barriers and solutions for the adoption and maintenance of healthy diet and physical activity among patients with coronary heart disease**

| **Barriers** | **Solutions** |
| --- | --- |
| **Barriers for the adoption and maintenance of healthy diet** | |
| 1. **Long-established habits** | - Comprehensive intervention |
| 1. **Healthy food is too expensive** | - Introduce healthy and affordable food |
| 1. **Stress or depression** | - Provide emotional-control strategies |
| 1. **Emotional eating such as anxiety, anger, bored** | - Provide information regarding physical activity as a diversional tactic |
| 1. **Hunger** | - Advise patients to have little food at each meal |
| 1. **Feeling deprived of food** | - Provide a specific, operable and appropriate culturally dietary plan |
| 1. **No time to prepare healthy food.** | - Send videos about making of simple meals |
| 1. **Lack of-motivation** | - Increase the motivation to change unhealthy diet |
| 1. **Can’t see the short-term effect** | - Provide interventions to increase effect perception |
| 1. **Lack of confidence** | - Provide interventions to increase self-efficacy |
| 1. **Lack of self-control and cannot resist temptation** | - Provide interventions to divert patients’ attention or reduce stimulus |
| 1. **Difficult to limit the proportion of meals per meal.** | - Provide an action plan for diet intake - Accept patients who occasionally fail to follow the diet plan |
| 1. **Lack of knowledge about healthy diet** | - Send knowledge test and provide correct answers |
| 1. **Thought medication treatment is enough** | - Explain the association between diet change and the mortality of CHD - Focus on the advantages and importance of diet change |
| 1. **Diligence and thrift and don’t want to waste food** | - Interpret the harm of food deterioration |
| 1. **Much high-fat, high-salt food around** | - Convey the importance of consuming low-fat and low-salt food - Invite family members or friends to change diet pattern together |
| 1. **Living with families** | - Invite family members or friends to change diet pattern together - Increase social support |
| 1. **Special festivals or holiday** | - Keep reminding that diet is important for health - Tell patients that they can control themselves even on important dinner occasions |
| 1. **Family members don’t know how to offer healthy food** | - Families and patients receive intervention together - Send information on healthy cooking to patients’ families |
| **Barriers for change and maintenance of physical inactivity** | |
| 1. **Low health literacy patients** | - Interventions should be simple |
| 1. **Depression, anxiety or stress** | - Information that physical activity can alleviate unhealthy emotions such as depression and anxiety. - Set a small, achievable goal - Increase social support |
| 1. **Safety** | - Provide suggestions regarding safety during physical activity (i.e. physical activities should be performed with partners indoors or in public places) |
| 1. **Limited time** | - Physical activities planning should be incorporated into daily activities according to his/her schedule - Provide information in terms of time management - Evaluate the energy expenditure of daily activities for sedentary patients. Emphasize that daily housework is also a moderate type of physical activities. |
| 1. **Lack of motivation** | - Provide motivational feedbacks for patients - Encourage the patient finding a partner to exercise together - Send individualized messages to patients rather than broadcasting in groups |
| 1. **Lack of social support** | - Send reminders to patients’ spouse or family members - Encourage family members to participate in projects and install the iCare Apps - Introduce nearby sports club to patients |
| 1. **Cost issue** | - Provide information about physical activities that do not need special equipment such as walking and dancing with subsidized financial aid |
| 1. **Bad weather** | - Provide information about indoor physical activities |
| 1. **Current health problems** | - Evaluate patients’ current health conditions, then provide an individualized guide for patients with appropriate type, duration and intensity of physical activities |
| 1. **Busy work schedule** | - Incorporate walking in the daily commute |
| 1. **Vacation or important festivals** | - Provide useful information that patient can invite his/her family members/friends to take part in physical activities during the vacation - Make a schedule for physical activity during vacation and provide reminders according to the schedule |
| 1. **Injury** | - Further assessment of patients’ health conditions, and provide information about bed rest, and rehabilitation therapy under the guidance of orthopedic doctor |
| 1. **Illness** | - Allow patients to temporarily reduce or pause their physical activities - Adjust short-term goals to overcome difficulties |
| 1. **Pain after exercising** | - Explain to patients why muscle soreness occurs after exercise. Provide information that the symptoms of muscle ache may disappear - Suggest patients to take analgesics before exercise - Provide physical therapy consultation |
| 1. **Lack of self-monitoring** | - Provide in-time reminder messages |
