# Supplementary material for: Development of interventions for an intelligent and individualized mobile health care system to promote healthy diet and physical activity: using an intervention mapping framework
Source: BMC Public Health. 2019 Oct 17;19:1311. doi: 10.1186/s12889-019-7639-7 (PMC6798431; doi:10.1186/s12889-019-7639-7)
Supplement: Supplementary file 6 — Additional file 6. Comic about the benefits of low salt and low fat. [file 12889_2019_7639_MOESM6_ESM.pptx]

## Slide 1
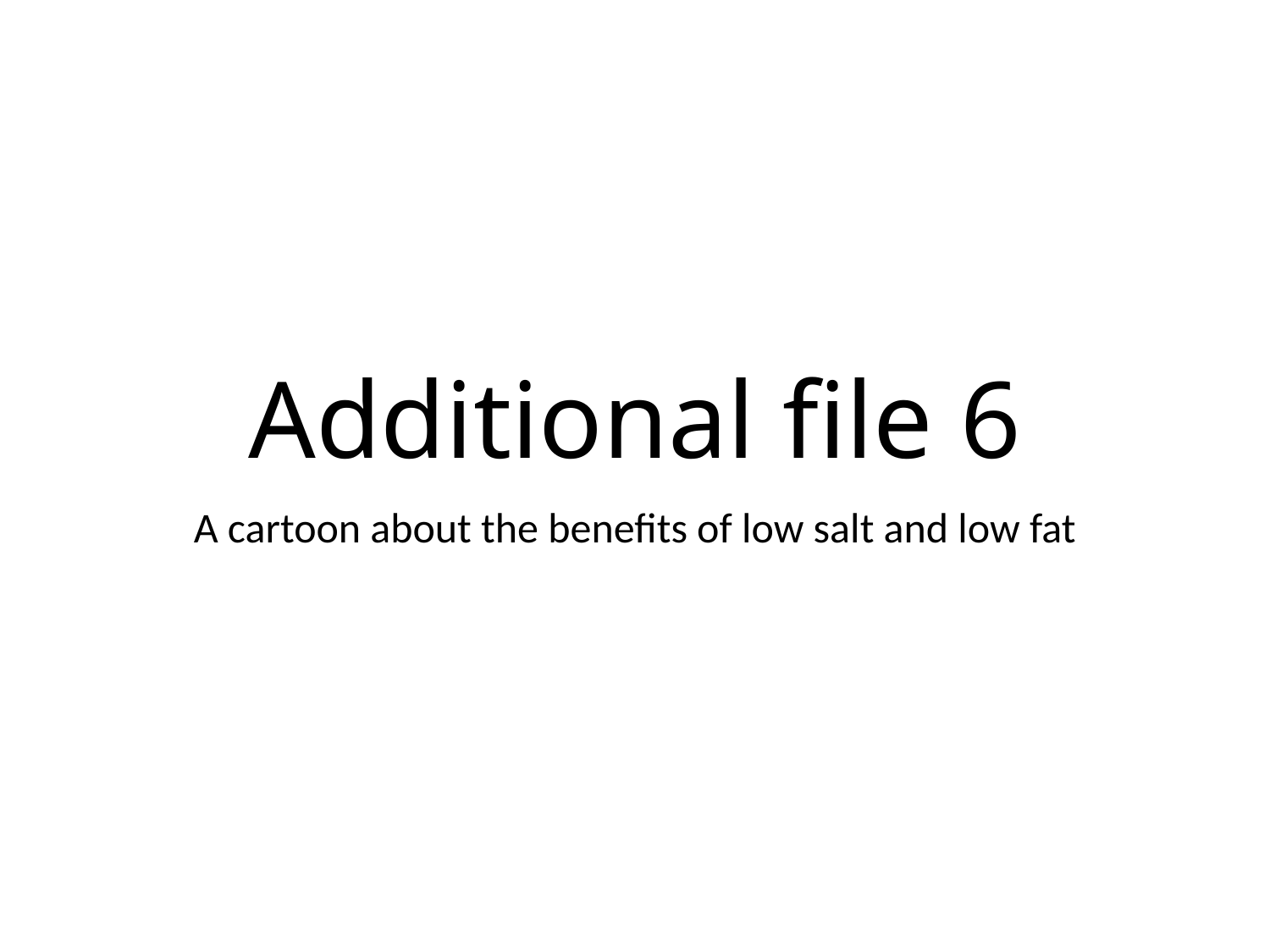

# Additional file 6
A cartoon about the benefits of low salt and low fat

## Slide 2
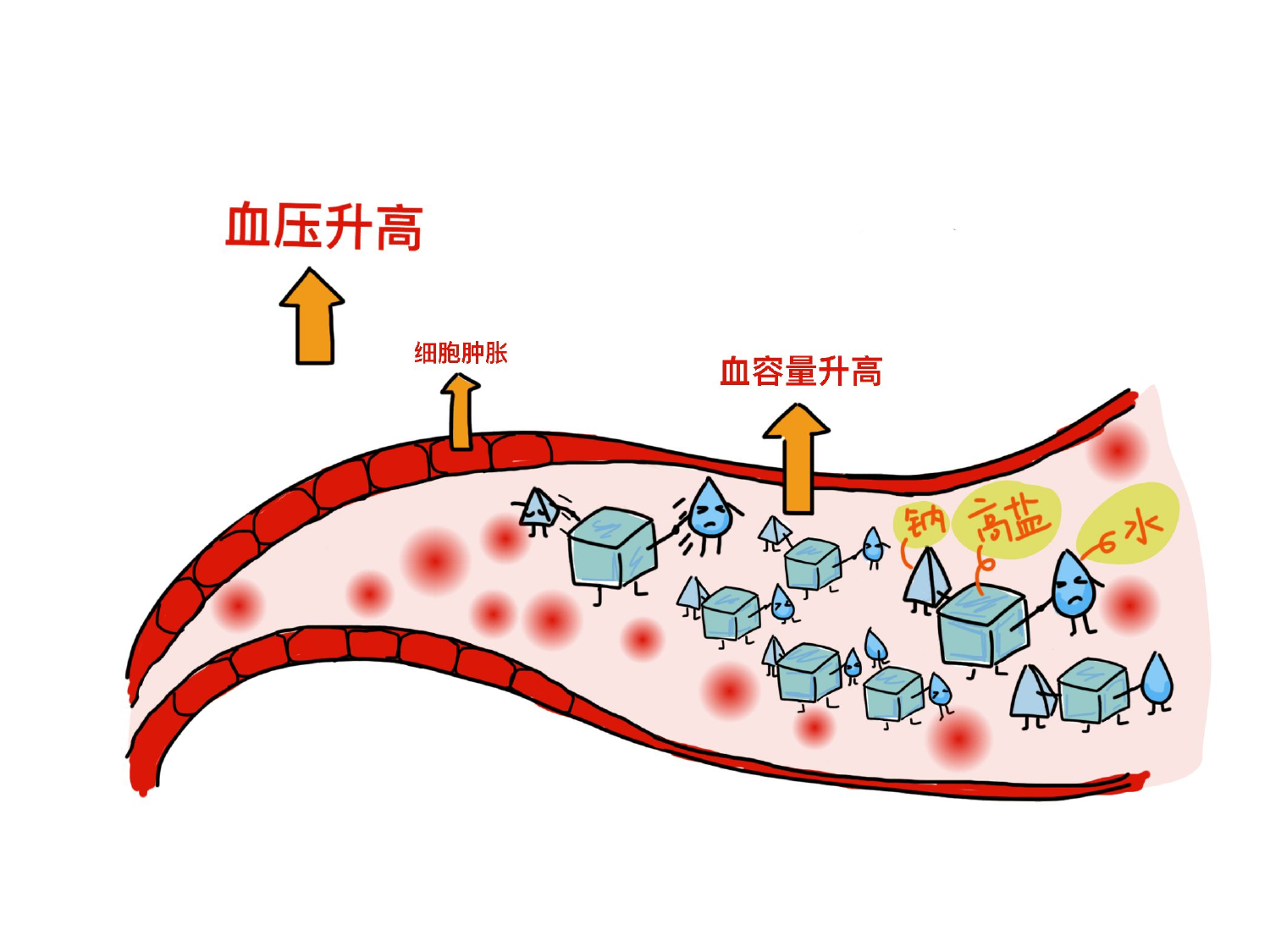

#

## Slide 3
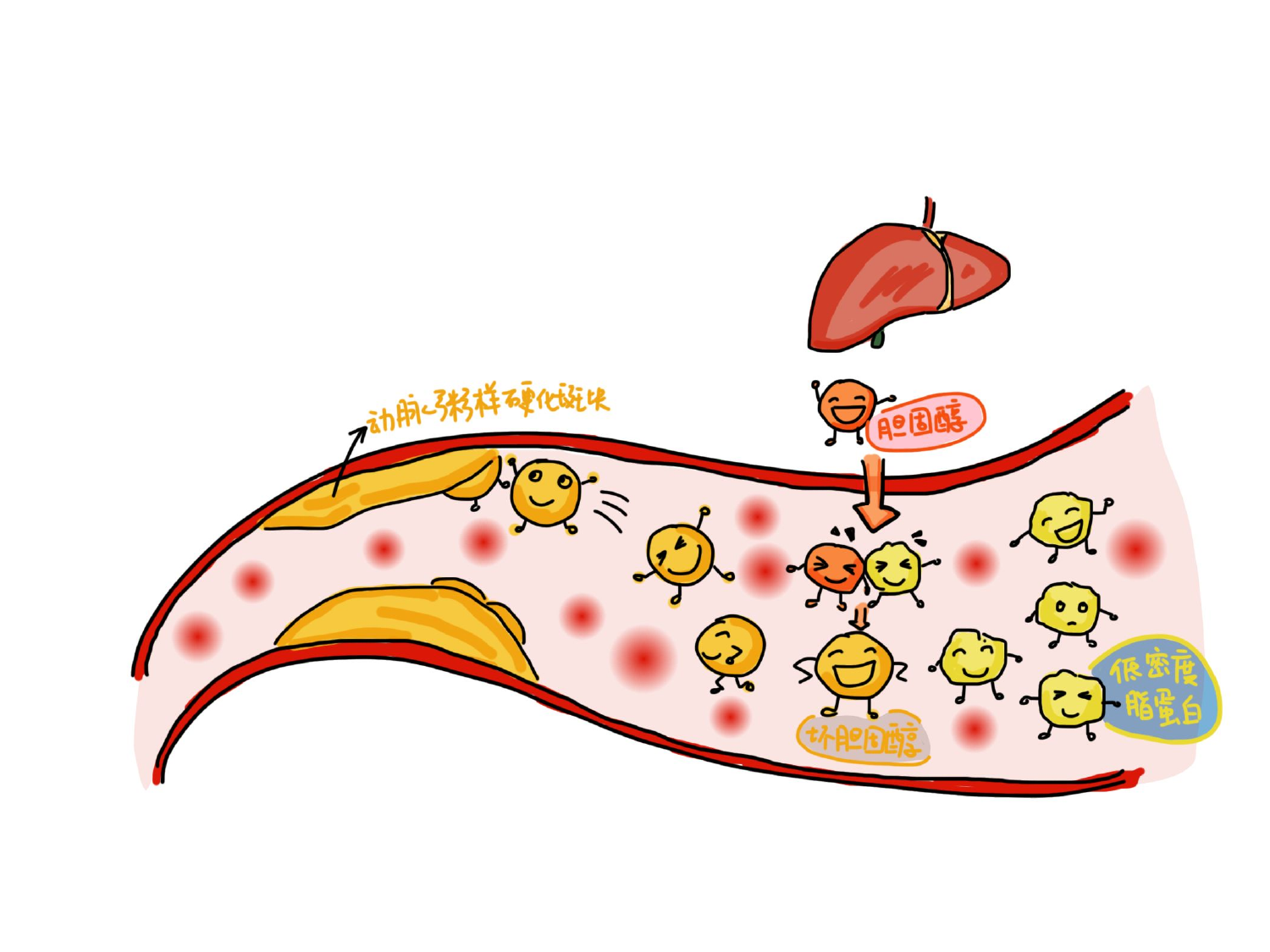

#

## Slide 4
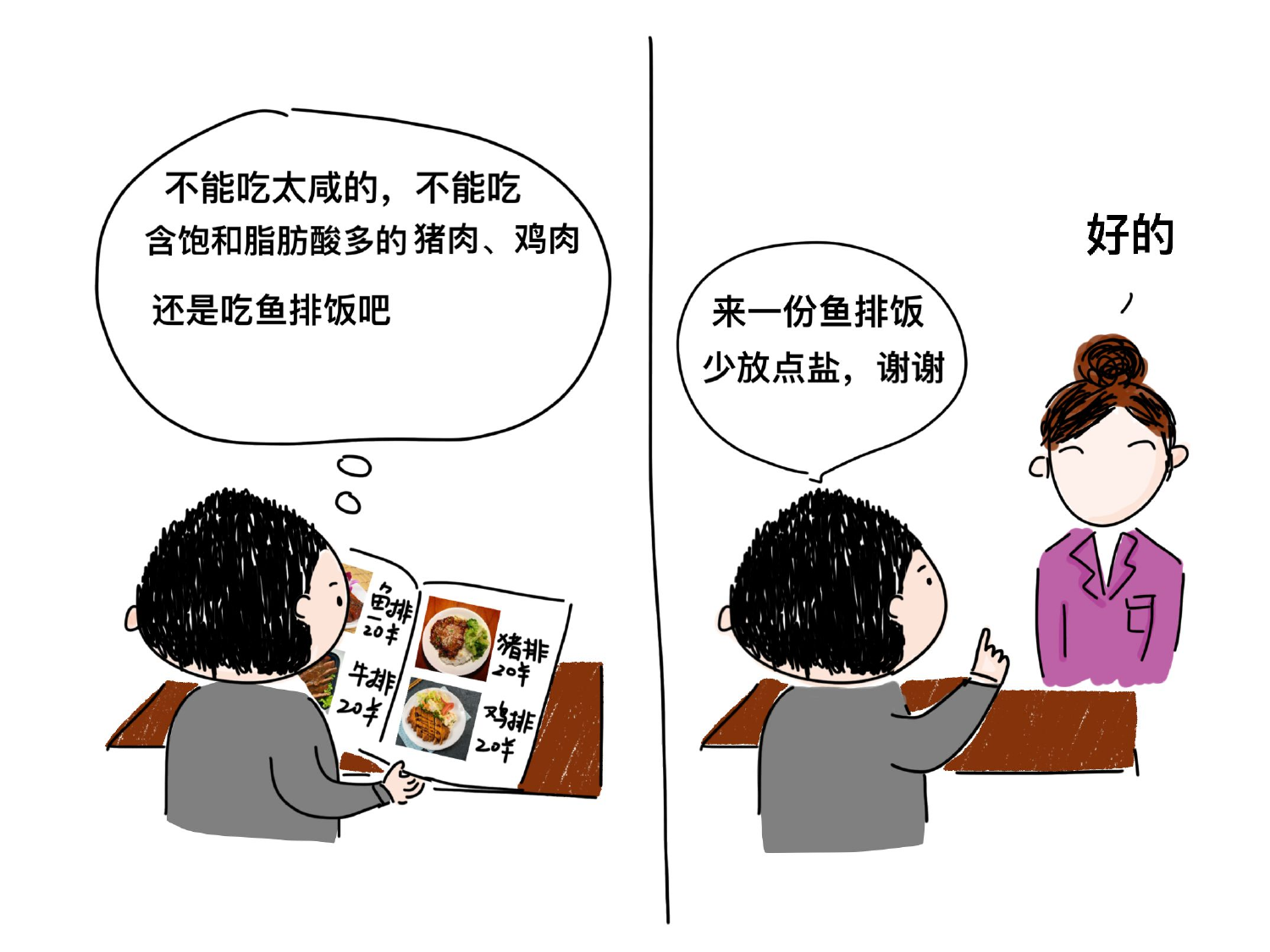

#

## Slide 5
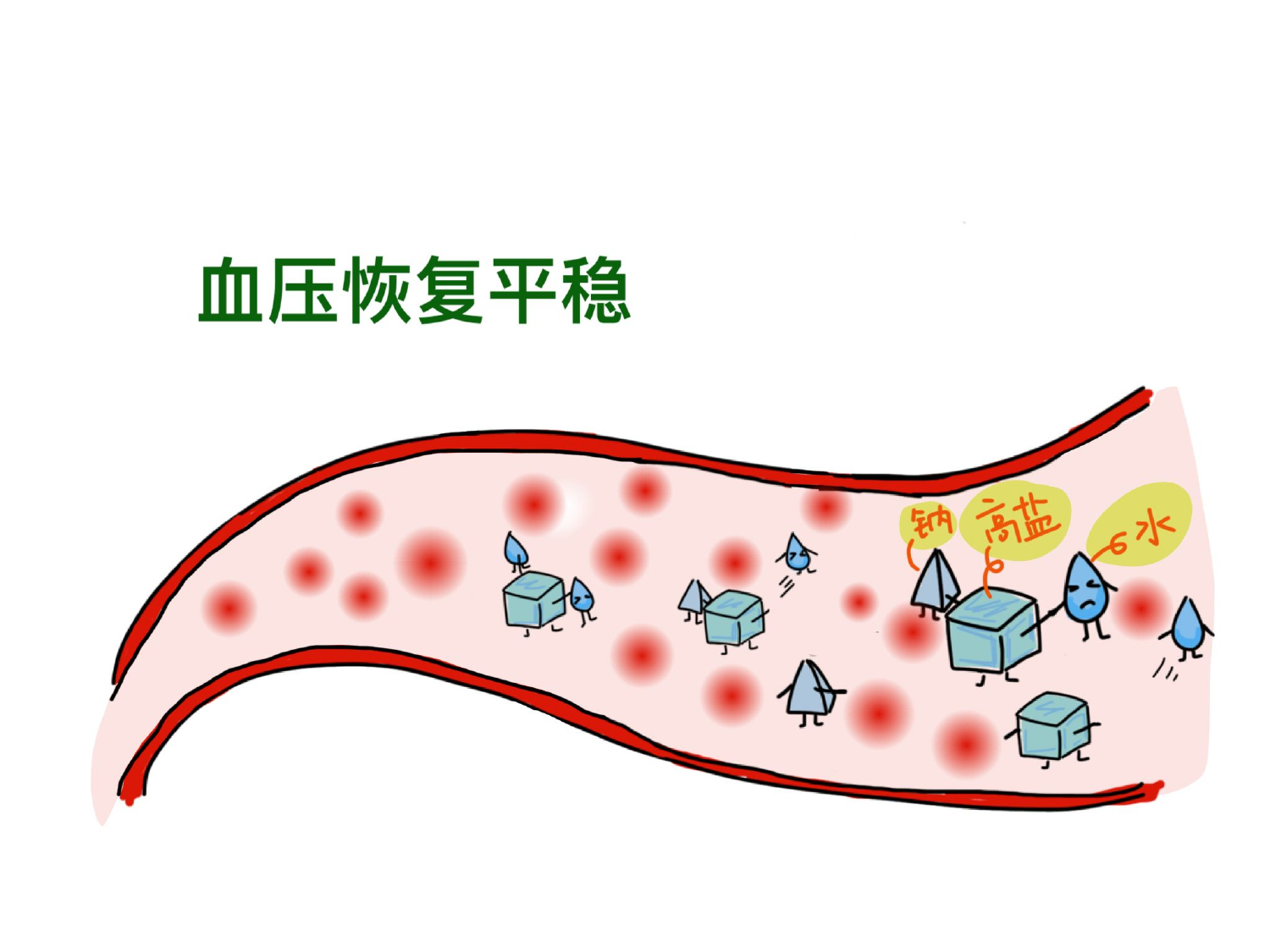

#

## Slide 6
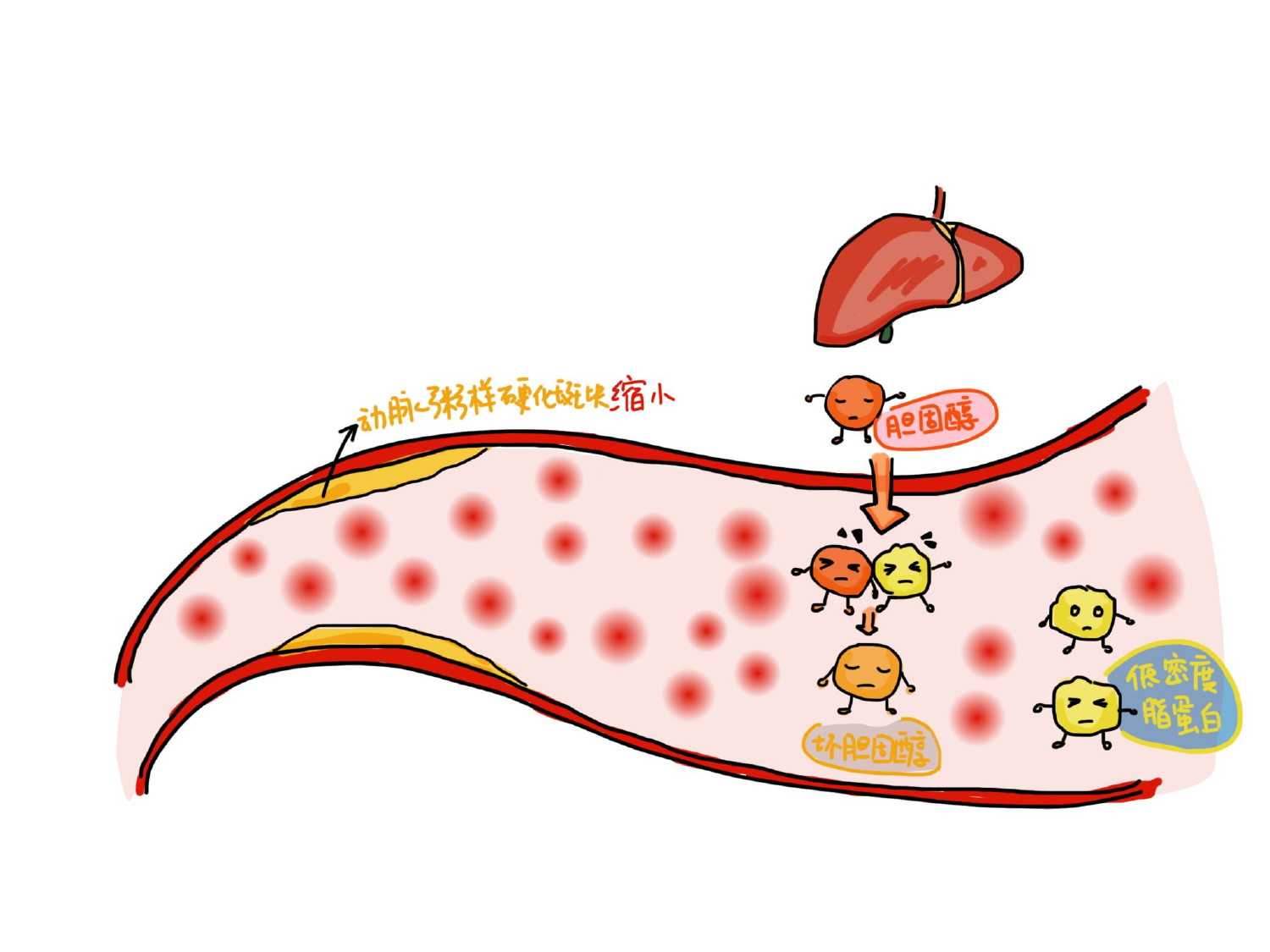

#

## Slide 7
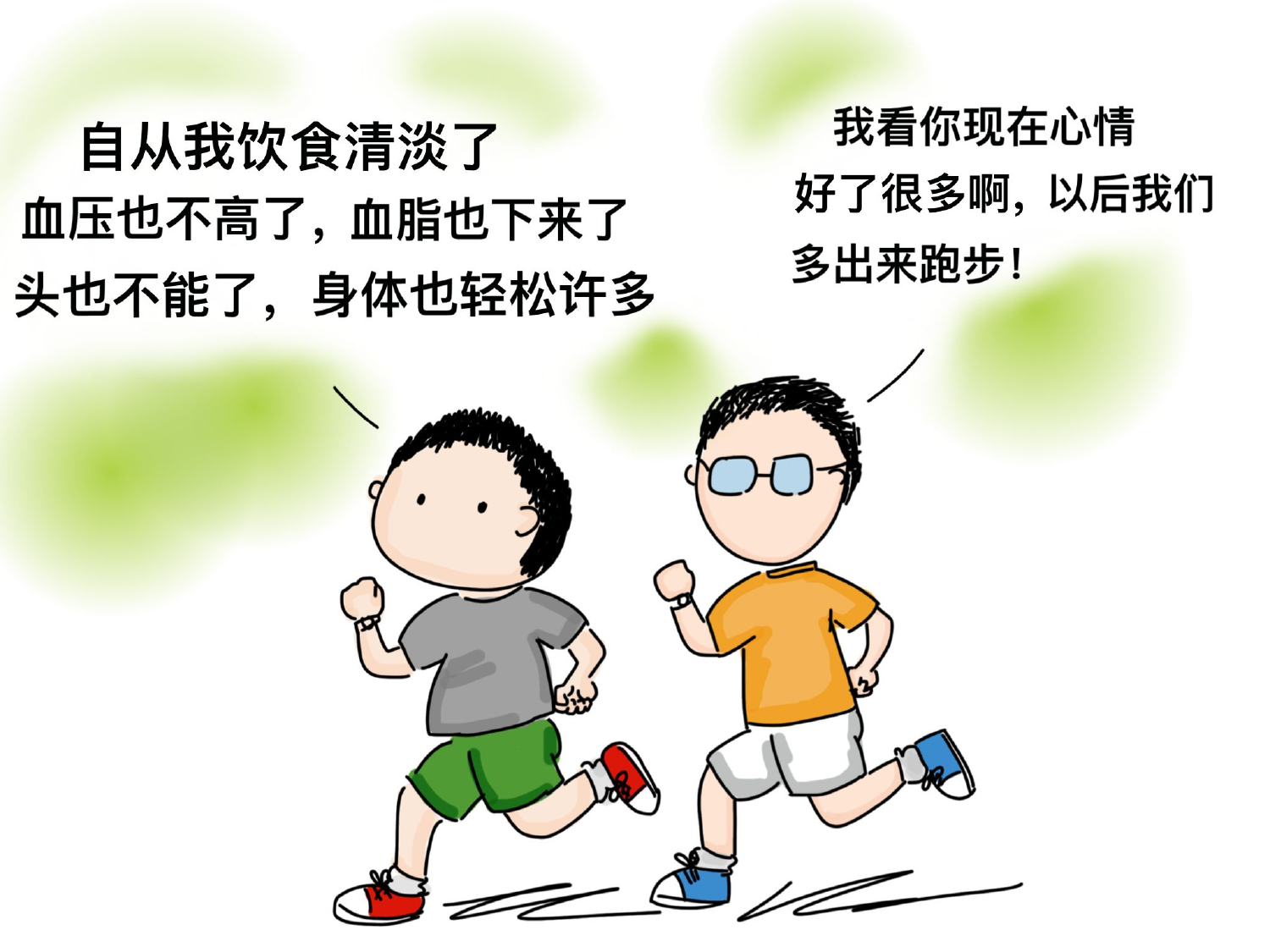

#
